# Effects of Disturbed Body Perception on Arm Function in Patients With Frozen Shoulder

**DOI:** 10.7759/cureus.69031

**Published:** 2024-09-09

**Authors:** Shinichi Matsumoto, Yuh Yamashita, Xiaoqian Chang, Takashi Hasegawa, Keita Nishi, Takefumi Moriuchi, Kaoru Noguchi, Yuichi Nakao, Keizo Furukawa, Toshio Higashi

**Affiliations:** 1 Department of Health Sciences, Graduate School of Biomedical Sciences, Nagasaki University, Nagasaki, JPN; 2 Department of Rehabilitation, Furukawa Miyata Orthopedic and Internal Medicine Clinic, Nagasaki, JPN; 3 Department of Rehabilitation, Morinaga Orthopedic Clinic, Saga, JPN; 4 Department of Rehabilitation, Wajinkai Hospital, Nagasaki, JPN; 5 Department of Physical Therapy, School of Health Science, Toyohashi Sozo University, Toyohashi, JPN; 6 Department of Occupational Therapy Science, Graduate School of Biomedical Sciences, Nagasaki University, Nagasaki, JPN; 7 Department of Orthopedics, Furukawa Miyata Orthopedic and Internal Medicine Clinic, Nagasaki, JPN

**Keywords:** arm function, disturbed body perception, freshaq, frozen shoulder, pain, pain-related beliefs

## Abstract

Purpose

Considering pain-related beliefs, this study aimed to investigate the influence of disturbed body perception on arm function in patients with frozen shoulder (FS).

Methods

This study included 90 patients with FS who visited the orthopedic outpatient clinic. We recorded measurements including the Visual Analog Scale (VAS) for pain at rest and during movement, the quick disability of the arm, shoulder, and hand (QDASH) questionnaire for perceived arm function, the short version of the Pain Self-Efficacy Questionnaire-2 (PSEQ-2) for self-efficacy in pain, the short version of the Pain Catastrophizing Scale (PCS-6) for catastrophic thoughts about pain, the short version of the Tampa Scale for Kinesiophobia (TSK-11) for fear of movement, and the Fremantle Shoulder Awareness Questionnaire (FreSHAQ) for disturbed perception around the shoulder.

For statistical analysis, we constructed three models. Model 1 used the QDASH score as the dependent variable, with gender, age, duration of disease onset, VAS score at rest, and VAS score during movement as control variables. Model 2 included pain-related beliefs (PSEQ-2, PCS-6, and TSK-11) added to the variables in Model 1. Model 3 further included the FreSHAQ to the variables in Model 2. We calculated the difference in the adjusted coefficients of determination (R² adj) from Model 1 to Model 2 and Model 3 to determine the amount of change (ΔR² adj). Additionally, we calculated the standardized regression coefficient (β-value) of the input factors to examine their effects.

Result

Hierarchical multiple regression analysis showed a ΔR² adj of 0.13 and 0.17 for Models 2 and 3, respectively, indicating an increase in ΔR²adj after adding the FreSHAQ. Furthermore, the FreSHAQ had the largest effect of all factors, with β = 0.27 (p<0.05).

Conclusions

The influence of disturbed body perception on perceived arm function in patients with FS was demonstrated, suggesting the importance of considering somatosensory factors in clinical practice.

## Introduction

Frozen shoulder (FS) is a condition that occurs without a specific cause and is characterized by a limited range of motion (ROM) in both active and passive movements, as well as pain at rest and during movement, which impairs activities of daily living (ADL) and quality of life (QOL) [1]. Although it is difficult to accurately estimate the prevalence of FS due to the lack of clear causes or strict diagnostic criteria, it is generally believed to affect 2%-5% of the general population [1,2]. Additionally, many patients experience a prolonged period between the onset of symptoms and recovery. Consequently, long-term arm dysfunction can occur due to increased muscle tone resulting from compensatory movements to avoid pain and compensate for the limited ROM [3-6].

Associations of pain-related beliefs in patients with FS

In many patients with musculoskeletal pain, worsening pain-related beliefs have been reported to adversely affect functional disability and recovery of QOL [7,8]. Pain processing issues and cognitive-psychological factors may influence perceived arm function in patients with FS [6,8,9]. Baets et al. reported that pain-related beliefs, such as fear of movement and self-efficacy in the context of pain, explained approximately 26% of the variance in perceived arm function among patients with FS [8]. This underscores the importance of assessing pain-related beliefs in clinical practice and highlights the need to assess pain-related symptoms from multiple perspectives, beyond just regional issues.

Musculoskeletal disorders and disturbed body perception

An increasing number of reports have focused on disturbed body perception in patients with musculoskeletal disorders, such as chronic low back pain, knee osteoarthritis, and chronic neck pain, suggesting a relationship between disturbed body perception, pain, and functional disability [10-13].

Sensory input from organs such as vision and proprioceptors are processed through higher-brain perceptual mechanisms to control bodily functions. Body perception is learned and modified through repeated perceptual processes [14]. The brain continually predicts future events based on sensory input and strives to minimize discrepancies between these predictions and actual sensory information. When predictions are accurate, prediction errors are minimal, and the brain's energy expenditure is efficient. Conversely, incorrect predictions lead to discrepancies in sensory information interpretation, which can alter pain perception. These erroneous predictions contribute to the reinforcement of chronic pain and disturbed body perception [12-15]. For instance, in phenomena such as the “Rubber Hand Illusion,” disturbances in body perception result from errors in the brain's integration of external visual information with internal predictions [16]. Additionally, patients with Complex Regional Pain Syndrome (CRPS) and chronic low back pain have been reported to experience body perception illusions, such as deteriorated two-point discrimination thresholds and distorted self-image [17,18]. These phenomena suggest that in the central somatosensory processing process, the brain is unable to accurately perceive painful areas, thereby exacerbating pain and dysfunction [19,20].

Disturbed body perception with FS

Delayed reaction times in mental rotation tasks and deteriorated two-point discrimination thresholds have been observed in patients with FS, which may be associated with disturbed body perception [21-23]. Furthermore, therapeutic interventions such as mirror therapy and visual feedback have been reported to alleviate symptoms in patients with FS [24-26]. This highlights the need for interventions that address regional joint pain and disturbed body perception.

Thus, while disturbed body perception is suggested to affect arm function in patients with FS, previous investigations have not adequately considered the impact of pain-related beliefs. Therapeutic interventions aimed at restructuring body perception can reduce pain and functional disability, indicating that disturbed body perception is an important therapeutic target when planning individualized rehabilitation [10,13,27,28]. Therefore, evaluating disturbed body perception in clinical practice is of great significance.

Purpose of the study

This study aimed to examine the relationship between disturbed body perception and cognitive-psychological factors in patients with FS, as well as to examine the influence of disturbed body perception on arm function in these patients, considering the impact of pain-related beliefs.

## Materials and methods

Participants in this study were adults with a clinical diagnosis of unilateral FS who visited the outpatient orthopedic clinic at Furukawa-Miyata Orthopedic and Internal Medicine Clinic between April 1, 2021 and March 30, 2024. The inclusion criteria were as follows: a) at least 25% limitation in ROM in at least two directions in the affected shoulder compared to the unaffected shoulder (flexion and external rotation measured with a goniometer, and internal rotation measured at the index finger); b) there had to be at least a 50% limitation in external rotation on the affected shoulder compared to the unaffected shoulder; c) the presence of pain and limited ROM for at least one month; and d) gradual appearance of symptoms, reaching a plateau, or worsening [4]. Exclusion criteria were as follows: previous surgical treatment for FS, autoimmune disease, trauma around the affected shoulder joint or upper extremity surgery within the past year, cognitive impairment that interfered with evaluation, and participation in sports activities involving the affected upper extremity [4].

Verbal and written consent for participation in the study was obtained from all participants. In accordance with the Helsinki Declaration, this study was approved by the Ethics Committee of Nagasaki University Graduate School of Biomedical Sciences (22090801) and was conducted with the utmost care to ensure the protection of participants.

Basic information, including age, gender, duration of pain onset, medical history, and current medical status, was obtained. Various questionnaires were used for assessment, namely, pain at rest and during movement using the Visual Analog Scale (VAS), upper limb function using the quick disability of the arm, shoulder, and hand (QDASH), self-efficacy using the shorted version of the Pain Self-Efficacy Questionnaire-2 (PSEQ-2), catastrophizing thoughts using the shorted version of the Pain Catastrophizing Scale (PCS-6), fear of movement using the shorted version of the Tampa Scale for Kinesiophobia (TSK-11), and disturbed body perception around the shoulder joint using the Fremantle Shoulder Awareness Questionnaire (FreSHAQ).

Pain VAS

Resting pain and pain during movement were measured using the VAS, where 0 indicated “no pain at all” and 100 indicated “the worst pain imaginable.”

Quick disability of the arm, shoulder, and hand

The QDASH is a shortened version of the disability arm elbow surgery (DASH) questionnaire, which measures the degree of upper extremity dysfunction. The DASH originally consisted of 30 items, while the QDASH consisted of 11 items. The QDASH comprises eight functional items, scored from 1 (no difficulty) to 5 (unable to perform activity), three pain-related items, and scored from 1 (no symptoms) to 5 (very severe symptoms) [29-31]. Scores are expressed as a percentage, with 0% indicating no disability and 100% indicating maximum disability. The QDASH is compatible with the full DASH version to a certain extent, allowing for comparative analysis [29-31].

Pain self-efficacy questionnaire (PSEQ-2)

The PSEQ-2 is a shortened, two-item version of the original 10-item PSEQ. PSEQ-2 measures self-efficacy related to pain and has been validated for both reliability and validity [32]. PSEQ-2 measures the degree of confidence in achieving specific behaviors or activities despite experiencing pain. Each item is rated on a scale from 0 (not at all confident) to 6 (completely confident), yielding a total score ranging from 0 to 12. Higher scores indicate greater self-efficacy concerning pain.

Pain catastrophizing scale (PCS-6)

The PCS-6 is a shortened, six-item version of the original 13-item PCS, designed to assess catastrophic thinking related to pain. The PCS-6 is validated for both reliability and validity [33]. PCS-6 consists of three subscales: “ruminating,” involving persistent thoughts about the pain; “helplessness,” reflecting feelings of being unable to do anything about the pain; and “magnification,” involving overestimation of the severity of the pain. Each item is measured on a scale from 0 (not at all) to 4 (all the time), resulting in a total score of 0 to 24.

Tampa scale for kinesiophobia (TSK-11)

The TSK-11 is an 11-item shortened version of the original 17-item Tampa Scale for Kinesiophobia. TSK-11 is used to assess fear and avoidance beliefs related to movement and has been confirmed to be both reliable and valid [34]. Each item is scored from 1 (strongly disagree) to 4 (strongly agree), with higher scores indicating stronger fear and avoidance beliefs about movement.

Disturbed body perception

The FreSHAQ was specifically developed to assess disturbed body perception in patients with shoulder disorders. FreSHAQ is based on the Fremantle Back Awareness Questionnaire, which is used to evaluate disturbed body perception in patients with low back pain and has been confirmed to be both reliable and valid [11,35]. The FreSHAQ consists of nine items related to neglect-like symptoms, reduced proprioceptive sensation, and perceived body part shape and size. Each item is rated on a scale from 0 (never) to 4 (always), yielding a total score ranging from 0 to 36. Higher FreSHAQ scores indicate greater disturbance in body perception [35].

Statistical analysis

Statistical analyses were performed using JMP Pro (17.0, SAS Institute Inc., Cary, USA) with a significance level of less than 5% (p<0.05). The Shapiro-Wilk test was used to test the normality of the data. Pearson and Spearman (normality or non-normality) correlation coefficients were calculated to investigate the association between each measure (age, duration, rest pain VAS, motion pain VAS, QDASH, PSEQ-2, PCS-6, TSK-11, and FreSHAQ).

To examine the influence of each factor on perceived arm function, univariate regression analysis was initially performed with QDASH as the dependent variable and each measure as the independent variable. Following this, hierarchical multiple regression analysis was conducted with QDASH as the dependent variable. The hierarchical multiple regression analysis was performed in the following steps: Model 1 was created with sex, age, duration, rest pain VAS, and motion pain VAS as the objective variables. Model 2 was created by adding PSEQ-2, PCS-6, and TSK-11 into Model 1. Model 3 was created by adding FreSHAQ to Model 2. To examine the effect of each factor, the change in adjusted R-squared (ΔR² adj) was calculated for Model 2 and Model 3 relative to Model 1 as the baseline. The β coefficients were compared to examine the impact of each factor. Assumptions for linear regression were verified.

## Results

Basic information and normality tests

Ninety participants (49 females (54.4％); mean age±SD: 56.7±8.7) were included in the analysis. Table 1 describes the basic attributes of the participants and the results of each questionnaire. The normality test indicated that only the TSK-11 data followed a normal distribution (p>0.05), while the other variables did not (p<0.05) (Table 1).

**Table 1:** Basic demographic and scores of the questionnaires Data are reported as the mean ± standard deviation or n (%). Duration, Duration of pain onset; VAS, Visual Analog Scale; QDASH, Quick Disability of the arm, shoulder, and hand; PSEQ-2, 2-item short version of the Pain Self-Efficacy Questionnaire; PCS-6, 6-item short version of the Pain Catastrophizing Scale; TSK-11, 11-item short version of the Tampa Scale for Kinesiophobia; FreSHAQ, Fremantle Shoulder Awareness Questionnaire

Variable	All cases (N=90)
Sex (female）	49 (54.4%)
Age (years)	56.7±8.7
Duration (months）	3.9±3.4
Pain-related factors questionnaire	
Rest pain VAS (0-100: mm)	16.2±21.4
Motion pain VAS (0-100: mm)	62.3±22.5
Q-DASH (0-100: point）	27.1±14.8
PSEQ-2 (0-12: point）	8.0±2.0
PCS-6 (0-18: point）	9.1±5.8
TSK-11 (11-44: point）	20.4±5.0
FrsSHAQ (0-36: point）	7.7±6.3

Results of the correlation analyses

Correlation analysis revealed no significant correlations between FreSHAQ and age or duration of onset. However, FreSHAQ showed significant correlations with resting pain VAS (ρ= 0.24, p<0.05), motion pain VAS (ρ= 0.23, p<0.05), QDASH (ρ＝0.49, p<0.01), PSEQ-2 (ρ＝-0.36, p<0.01) PCS-6 (ρ＝0.44, p<0.01), and TSK-11 (r＝0.45, p<0.01) (Table 2).

**Table 2:** Correlation matrix indicating the relation between control variables date and pain-related questionnaires *, p＜0.05; **, p＜0.01; Duration, Duration of pain onset; VAS, Visual Analog Scale; QDASH, Quick Disability of the Arm, Shoulder, and Hand; PSEQ-2, 2-item short version of the Pain Self-Efficacy Questionnaire; PCS-6, 6-item short version of the Pain Catastrophizing Scale; TSK-11, 11-item short version of the Tampa Scale for Kinesiophobia; FreSHAQ, Fremantle Shoulder Awareness Questionnaire

	Duration	Rest pain	Motion pain	QDASH	PSEQ-2	PCS-6	TSK-11	FreSHAQ
Age	-0.27*	-0.16	-0.22*	-0.24*	0.11	-0.18	-0.15	-0.00
Duration		0.04*	0.21*	0.27**	-0.09	0.09	0.12	0.13
Rest pain			0.32**	0.49**	-0.32**	0.27**	0.14	0.24*
Motion pain				0.42**	-0.15	0.37**	0.19	0.23*
QDASH					-0.48**	0.48**	0.35**	0.49**
PSEQ-2						-0.36**	-0.40**	-0.36**
PCS-6							0.61**	0.44**
TSK-11								0.45**

Results of the regression analyses

Univariate regression analysis demonstrated that pain-related beliefs were significantly associated with QDASH in the following ways: Sex(β=-0.20; p=0.06), Age(β=-0.16, p=0.12), Duration(β=0.20; p=0.06), PSEQ-2 (β=-0.48; <0.01), PCS-6 (β=0.54; <0.01), TSK-11 (β=0.37; <0.01), and FreSHAQ (β=0.55; <0.01) (Table 3).

**Table 3:** Hierarchical Regression model with perceived arm function explained by control variables and pain-related questionnaire in persons with frozen shoulder R², coefficient of determination; R² adj, R² adjusted value; ΔR² adj, Model X (R² adj)– Model-1 (R² adj) *, p＜0.05; **, p＜0.01; β, Standard Regression Coefficient Duration, Duration of pain onset; VAS, Visual Analog Scale; QDASH, Quick Disability Arm Shoulder Hand; PSEQ-2, 2-item short version of the Pain Self-Efficacy Questionnaire; PCS-6, 6-item short version of the Pain Catastrophizing Scale; TSK-11, 11-item short version of the Tampa Scale for Kinesiophobia; FreSHAQ, Fremantle Shoulder Awareness Questionnaire

	Univariable	Multivariable		
		Model-1	Model-2	Model-3
		R²=0.34，R²adj＝0.30	R²＝0.48，R²adj＝0.43, ΔR² adj=0.13	R²=0.53, R²adj＝0.47, ΔR² adj=0.17
	β	β	β	β
Sex( female)	-0.20	-0.17	-0.13	-0.08
Age(years)	-0.16	-0.03	0.00	-0.02
Duration(months)	0.20	0.04	0.07	0.08
Rest Pain VAS	0.46**	0.38**	0.25**	0.19*
Motion Pain VAS	0.39**	0.26**	0.14	0.12
PSEQ-2	-0.48**		-0.22*	-0.19*
PCS-6	0.54**		0.25*	0.22*
TSK-11	0.37**		0.06	-0.01
FreSHAQ	0.55**			0.27**

Results of hierarchical multiple regression analysis

The results of the hierarchical multiple regression analysis are as follows: Model 1; R² adj= 0.30, Model 2; R² adj=0.43 (PSEQ-2; β=-0.22, p<0.05; PCS-6; β=0.25, p<0.05; TSK-11; β=0.06, p=0.57 ), Model 3; R² adj=0.47 ( PSEQ-2; β=-0.19; p<0.05, PCS-6; β= 0.22; p<0.05, TSK-11; β=-0.01; p=0.90, FreSHAQ; β=0.27; p<0.01). The change in adjusted R² was 0.13 for Model 2 and 0.17 for Model 3, indicating that the addition of FreSHAQ improved the explanation of perceived arm function (Table 3). Additionally, Model 3 demonstrated significant relationships for all factors except TSK-11, with FreSHAQ having the largest β-value among the input factors.

## Discussion

The results of this study indicate that disturbed body perception, as measured by FreSHAQ, significantly influences perceived arm function in patients with FS. Model 3 (ΔR² adj=0.17), which included FreSHAQ, demonstrated a greater contribution to explaining perceived arm function compared to Model 2 (ΔR² adj=0.13), suggesting a greater influence of disturbed body perception. Comparison of the β-values for significant factors demonstrated that disturbed body perception (FreSHAQ; β=0.27) had a stronger effect than pain-related beliefs (PSEQ-2;β= -0.19, PCS-6; β=0.22) (Table 3).

Association between disturbed body perception and pain-related beliefs

The brain predicts events based on sensory input and aims to minimize the discrepancy between its predictions and the actual sensory information. When there is a large prediction error, discrepancies in the interpretation of sensory information can occur, potentially leading to pain and functional impairment [10,13-15,27,28]. The FreSHAQ is a straightforward and useful questionnaire to evaluate such somatosensory disturbances [35].

The FreSHAQ provides a comprehensive evaluation of somatosensory disturbances around the shoulder joint by assessing three perspectives: neglect-like symptoms, reduced kinesthetic acuity, and perceived body shape and size [35]. Reports indicate that while FreSHAQ is associated with pain intensity, it is not significantly related to the duration of pain onset [36]. Additionally, the level of disturbed body perception, as measured by FreSHAQ, is generally lower in patients with shoulder disorders compared to those with chronic low back pain (assessed by the Fremantle Back Awareness Questionnaire) or knee osteoarthritis (assessed by the Fremantle Knee Awareness Questionnaire) [11,35,37].

In the current study, the FreSHAQ score was 7.7 points, which is lower than the score reported by Nishigami et al., suggesting that the degree of disturbed body perception in patients with FS was not severe [35]. This finding remains controversial, as Nishigami et al.’s study included patients with chronic shoulder pain from various diagnoses other than FS, potentially influencing the results due to differing patient backgrounds. Correlation analysis revealed no significant relationship between disturbed body perception and the duration of pain onset. However, significant correlations were observed between pain intensity, functional impairment, catastrophic thoughts, and pain-related fear, which supports the previous report (Table 2) [11,35-38].

Effects of disturbed body perception on arm function

This study is the first to analyze the effects of disturbed body perception on arm function in patients with FS while considering the influence of pain-related beliefs. Previous reports have suggested that factors such as fear of movement, self-efficacy, and catastrophic thinking affect upper limb function in patients with FS [8,9]. The findings of this study suggest that disturbed body perception has the most significant effect on arm function in patients with FS, even when accounting for pain-related beliefs including self-efficacy (β=-0.19, p<0.05), fear of movement (β=-0.01, p=0.90), and catastrophic thinking (β=0.22, p<0.05) under pain (β =0.27, p<0.01) (Table 3) [8,9].

Multivariate analysis demonstrated that fear of movement was not significantly related to arm function in patients with FS, which contrasts with findings from previous studies (p=0.83). Additionally, the mild degree of upper extremity dysfunction in our study (27.1±14.8 points) compared to the QDASH score of 41.1±18.7 reported by Baets et al. may have influenced the background of the patients [8]. Moreover, while previous reports recruited patients from multiple orthopedic outpatient clinics and outpatient physical therapy facilities, this study recruited patients at the time of their initial physical therapy evaluation in patients with FS who visited a single primary care facility, an orthopedic outpatient clinic. Therefore, the fact that the evaluation was conducted before physical therapy intervention may have had a certain influence on the background factors of the patients, but this cannot be mentioned in this study.

Deteriorations in mental rotation tasks and two-point discrimination thresholds, which are believed to be related to disturbed body perception, have been observed in patients with FS. Additionally, the effectiveness of therapeutic interventions aimed at reconstructing somatosensory perception, such as mirror therapy and visual feedback, has been suggested [21-26]. However, these reports did not account for pain-related beliefs or the degree of disturbed body perception in their evaluations, leading to an insufficient evidence base.

While further validation through larger and prospective studies is needed, the current findings provide valuable insight into the role of disturbed body perception as a potentially significant factor. Although the mechanisms of pain, dysfunction, and somatosensory perception are not fully understood, therapeutic interventions appear to reduce these disturbances and should be emphasized in treatment [13]. Healthcare providers involved in the treatment of FS should consider the presence of disturbed body perception during evaluation and treatment planning.

Limitations

The causal relationship between disturbed body perception and arm function cannot be established due to the cross-sectional design of this study. In addition, the study was limited to a questionnaire and did not assess psychological status. Therefore, it may not have adequately assessed the patient's condition. Furthermore, a quantitative assessment of the pain processing involved in the chronicity of pain is not yet available. Future studies should also incorporate psychological tests for anxiety and depression, as well as quantitative sensory testing, to evaluate patients from multiple perspectives. Since the FreSHAQ values for the participants in this study were relatively low, we were unable to explore differences in outcomes by grouping participants according to the severity of their symptoms.

## Conclusions

This is the first report to investigate the influence of disturbed body perception on arm function in patients with FS while considering the influence of pain-related beliefs. The findings of this study suggest that disturbed body perception significantly affects arm function in these patients. Although further multifaceted validation through longitudinal studies, causal studies, and quantitative testing is needed, this study highlights the importance of incorporating somatosensory perception considerations in clinical practice and therapeutic interventions.
